# Supplementary material for: A C-terminal cytoplasmic retention motif and nuclear localization signal regulates nuclear import of TP53INP2
Source: J Cell Sci. 2025 Dec 19;138(24):jcs264267. doi: 10.1242/jcs.264267 (PMC12752491; doi:10.1242/jcs.264267)
Supplement: Supplementary information [file joces-138-264267-s1.pdf]

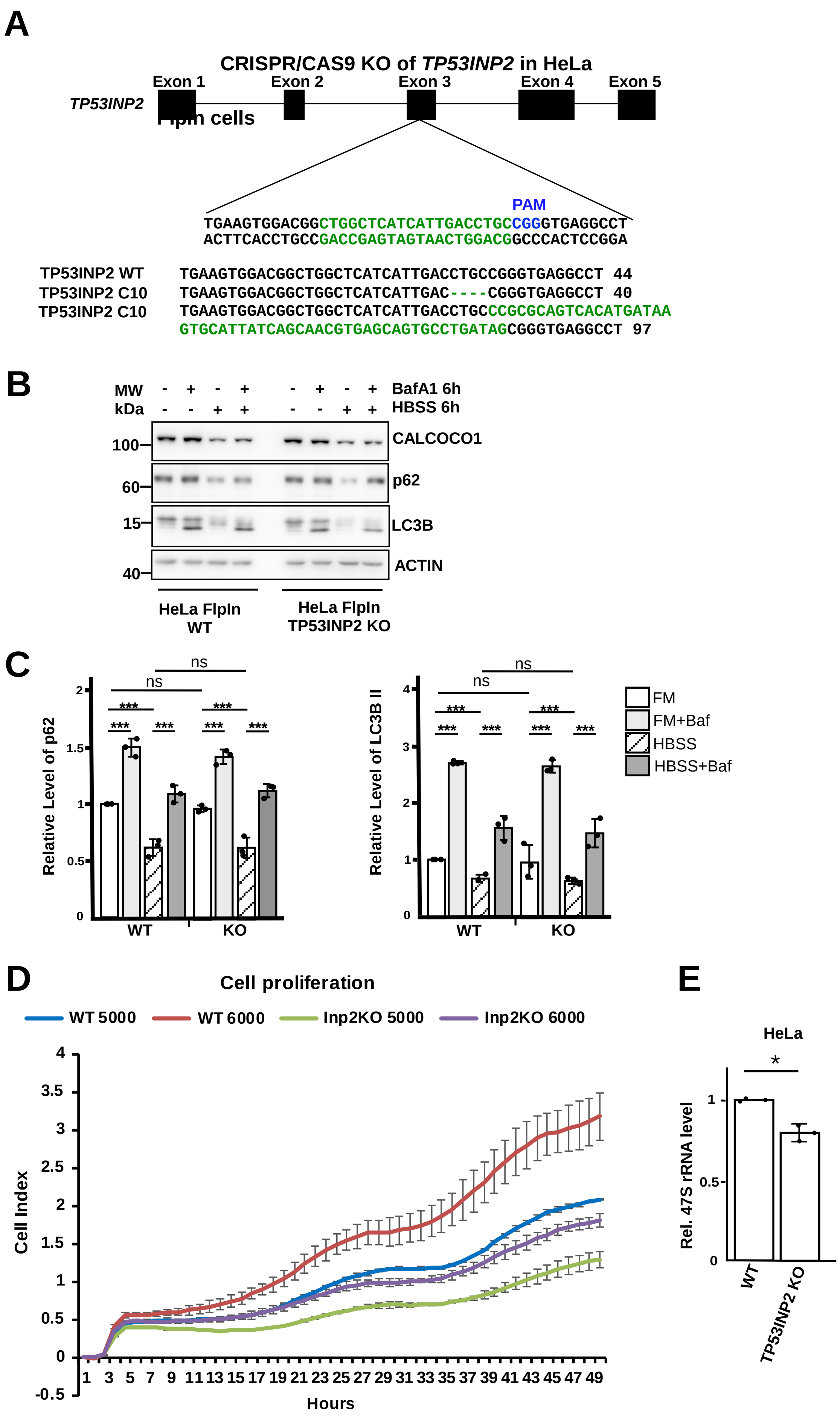

Journal of Cell Science • Supplementary information

Fig. S1. Generation of CRISPR/CAS9-mediated HeLa FlpIn TP53INP2 KO cells.

(**A**) Genomic sequences of indicated TP53INP2 exons targeted by guide RNAs. Shown are the sequences of two mutated alleles in TP53INP2 clone 10. More than 10 sequences were analyzed, and only two different alleles were identified. (**B and C)** Western blot analysis of p62, CALCOCO1, and LC3B in lysates from WT and TP53INP2 KO cells treated or not with HBSS and/or Bafilomycin A1 (BafA1) as indicated. The levels of LC3 and p62 were quantified by ImageJ. The bars represent the mean ± SD of band intensities relative to the actin loading controls. The statistical comparison was analyzed by one-way ANOVA followed by Tukey multiple comparison test and significance displayed as ***P < 0.001, *P < 0.01; ns is not significant. (**D**) Proliferation curves of WT and TP53INP2 KO cell lines (number of analyzed cells seeded are indicated). The diagram represents one of three biological replicates. (**E**) Relative level of 47S rRNA in WT and TP53INP2 KO cell lines.

Journal of Cell Science • Supplementary information

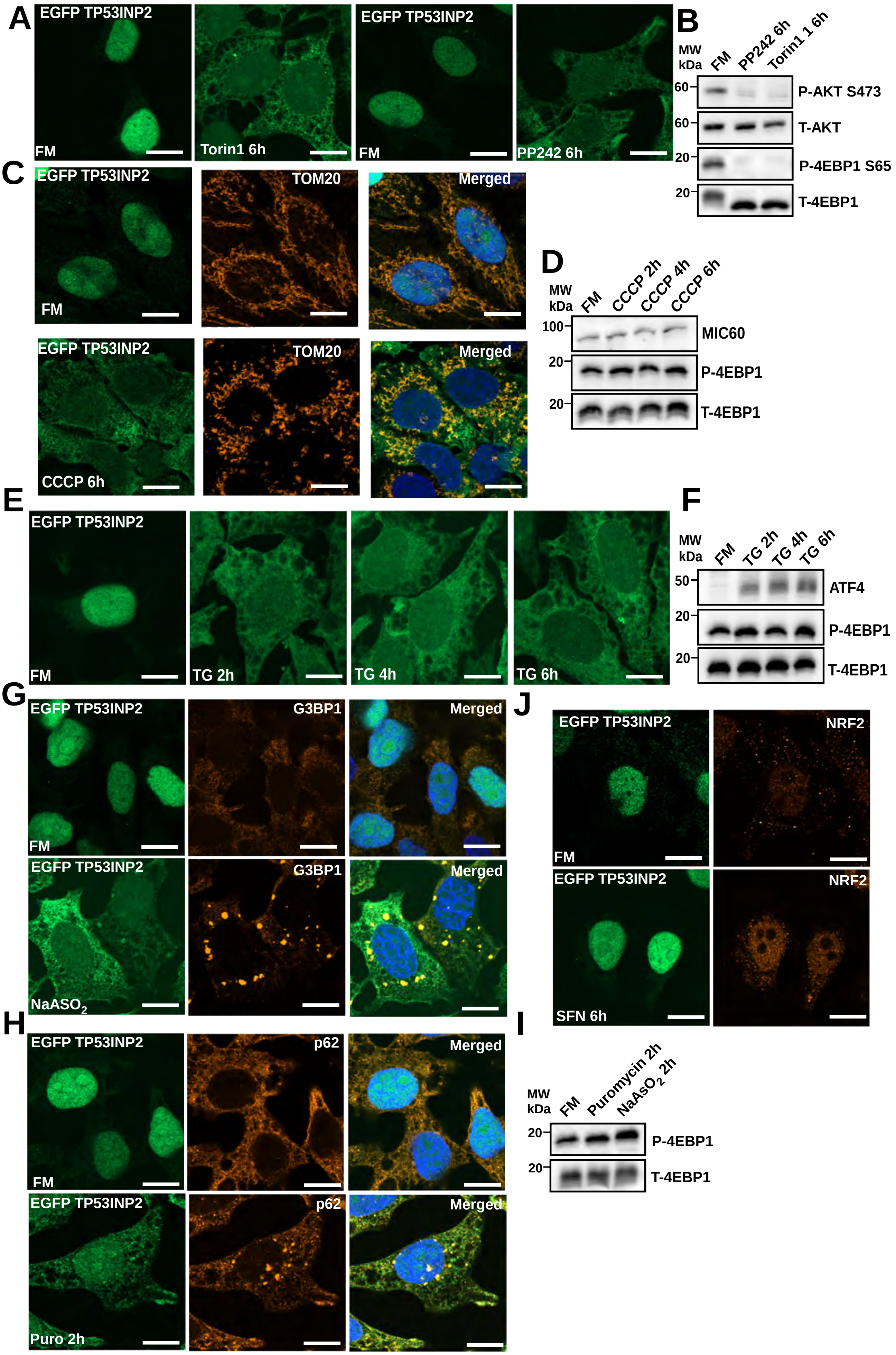

Journal of Cell Science • Supplementary information

Fig. S2. Various stressors lead to cytoplasmic redistribution of TP53INP2.

(A) Confocal images of HeLa FlpIn EGFP TP53INP2 cells treated with mTOR inhibitors Torin 1 and PP242 for 6 hours. Scale bars, 10 µm. **(B)** Western blot analysis of total and phosphorylated 4E-BP1 and AKT proteins in lysates from control and Torin 1/PP242 treated cells. **(C)** Confocal microscopy of HeLa FlpIn EGFP TP5INP2 cells treated with 10µM CCCP for 6 hours and immunostained with TOM20 antibody. Scale bars, 10 µm. **(D)** Immunoblotting of mTOR substrate 4E-BP1 (total and phosphorylated serine 65) from the cell lysate of control sample and CCCP treated sample. **(E)** Confocal microscopy of HeLa FlpIn EGFP TP53INP2 cells treated with Thapsigargin for 2hrs, 4hrs, and 6hrs. Scale bars, 10 µm. **(F)** Immunoblotting of ATF4 and 4-EBP1(total and phosphorylated serine 65) from control and thapsigargin treated cell lysate. **(G)** Confocal microscopy of Hela FlpIn EGFP TP53INP2 cells were treated with 50µM Sodium Arsenite for 2 hours and immunostained with G3BP1 antibody. Scale bars, 10 µm. **(H)** Confocal microscopy of HeLa FlpIn EGFP TP53INP2 treated with 2µg/ml of puromycin for 2 hours and immunostained with p62 antibody. Scale bars, 10 µm. **(I)** Western blot analysis of total and phosphorylated 4E-BP1 from control and sodium arsenite/puromycin treated sample. **(J)** Confocal microscopy of HeLa FlpIn EGFP TP53INP2 cells treated with 20µM SFN for 6 hours and immunostained with NRF2 antibody. Scale bars, 10 µm.

Journal of Cell Science • Supplementary information

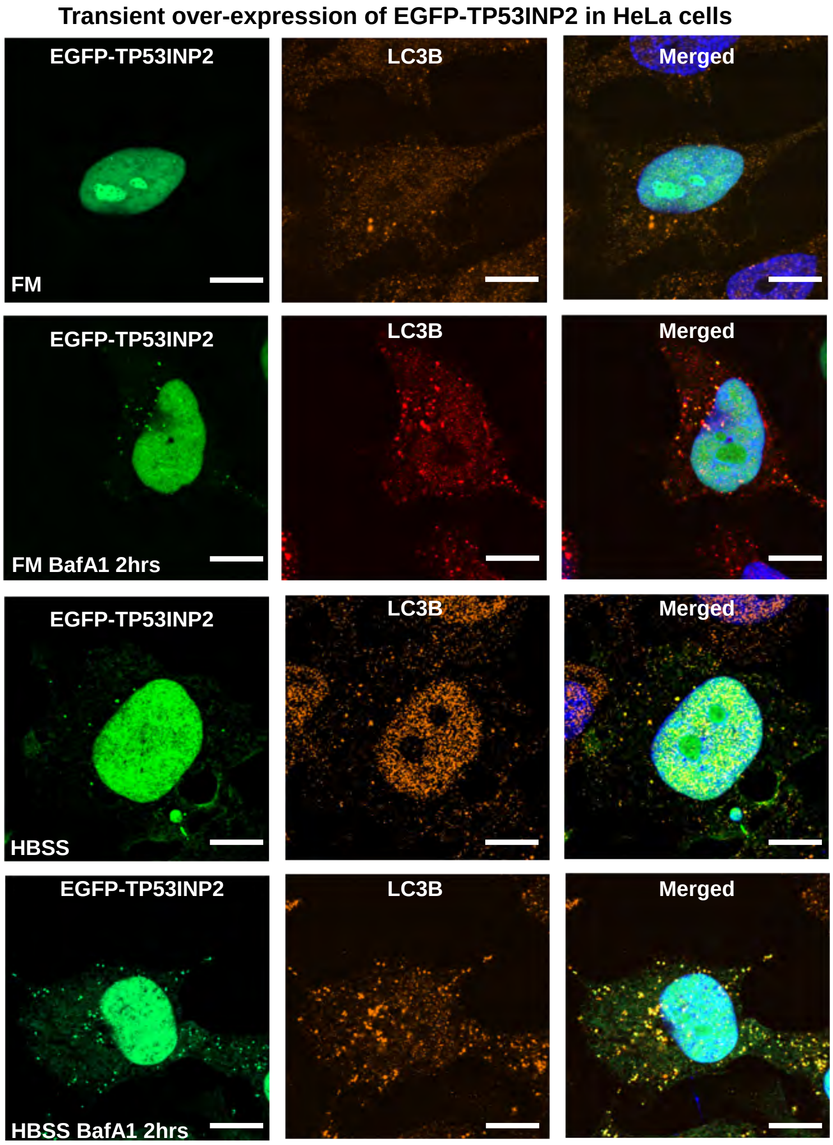

Fig. S3. Overexpression of TP53INP2 by transient transfection impairs its normal subcellular localization pattern.

Upon transient transfection over-expressed EGFP-TP53INP2 formed puncta that co-localized with LC3B in the cytoplasm upon starvation and accumulated in response to BafA1 treatment. HeLa FlpIn cells transiently transfected with pDest EGFP-TP53INP2 expression plasmids were subjected to the indicated treatments and analyzed by confocal fluorescence microscopy 24 h after transfection. Endogenous LC3B was detected with LC3B antibodies. Scale bars, 10 µm.

Journal of Cell Science • Supplementary information

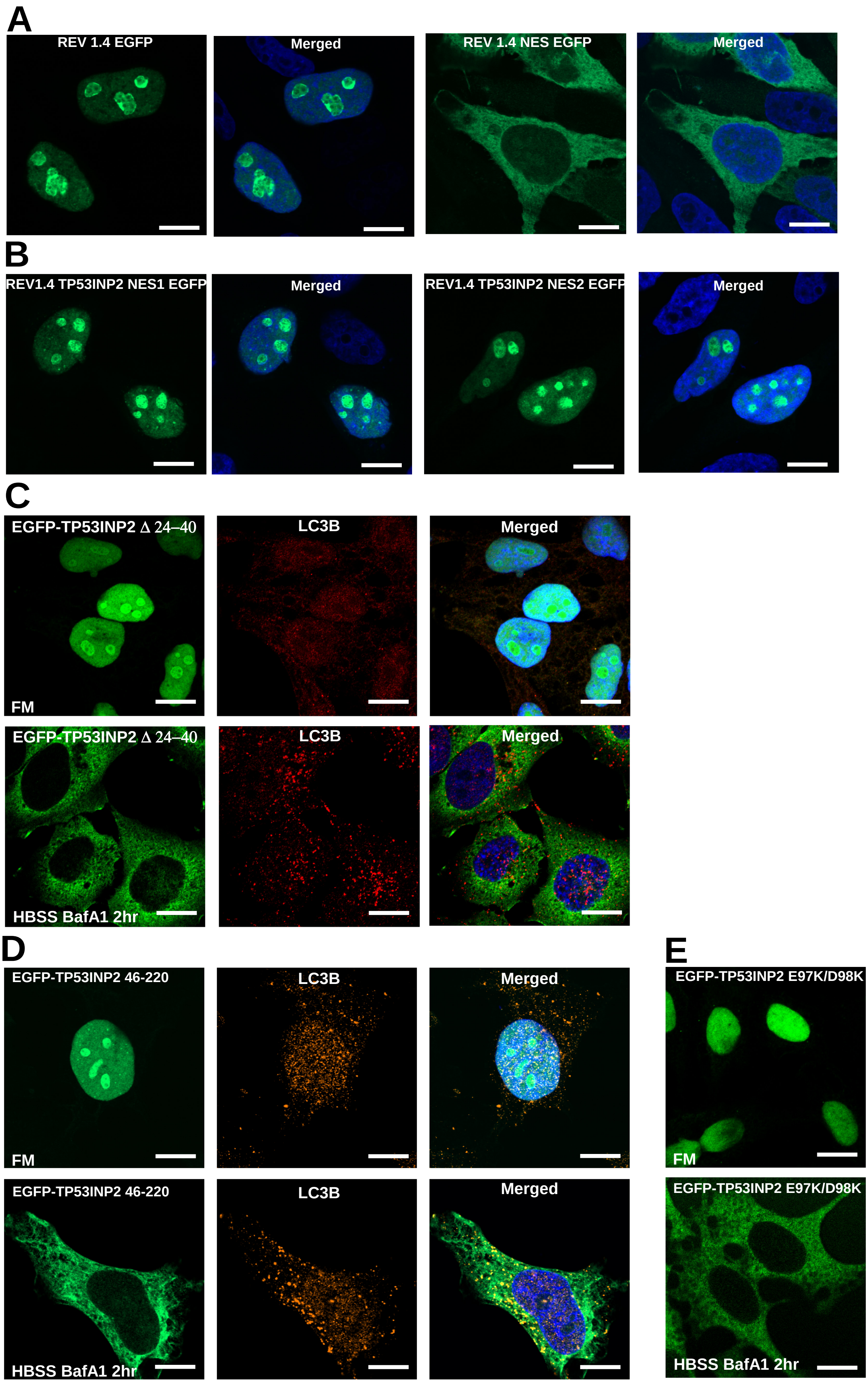

Journal of Cell Science • Supplementary information

Fig. S4. The cytoplasmic localization of TP53INP2 upon starvation is independent of its LIR and predicted NES motifs.

(A and B) HeLa cells were transfected with the indicated REV-EGFP constructs and analyzed by confocal fluorescence microscopy. REV1.4 EGFP is a negative control without any NES motif. REV1.4 NES EGFP is a positive control containing the NES from the REV protein. (**C and D)** HeLa FlpIn TP53INP2 KO cells stably reconstituted with the indicated EGFP-TP53INP2 constructs were treated as indicated, stained with LC3B antibodies, and analyzed by confocal fluorescence microscopy. Scale bars, 10 µm. **(E)** Confocal microscopy of Hela FlpIn TP53INP2 KO cells expressing EGFP-TP53INP2 E97K/D98K grown either in Full medium or HBSS. Scale bars, 10 µm.

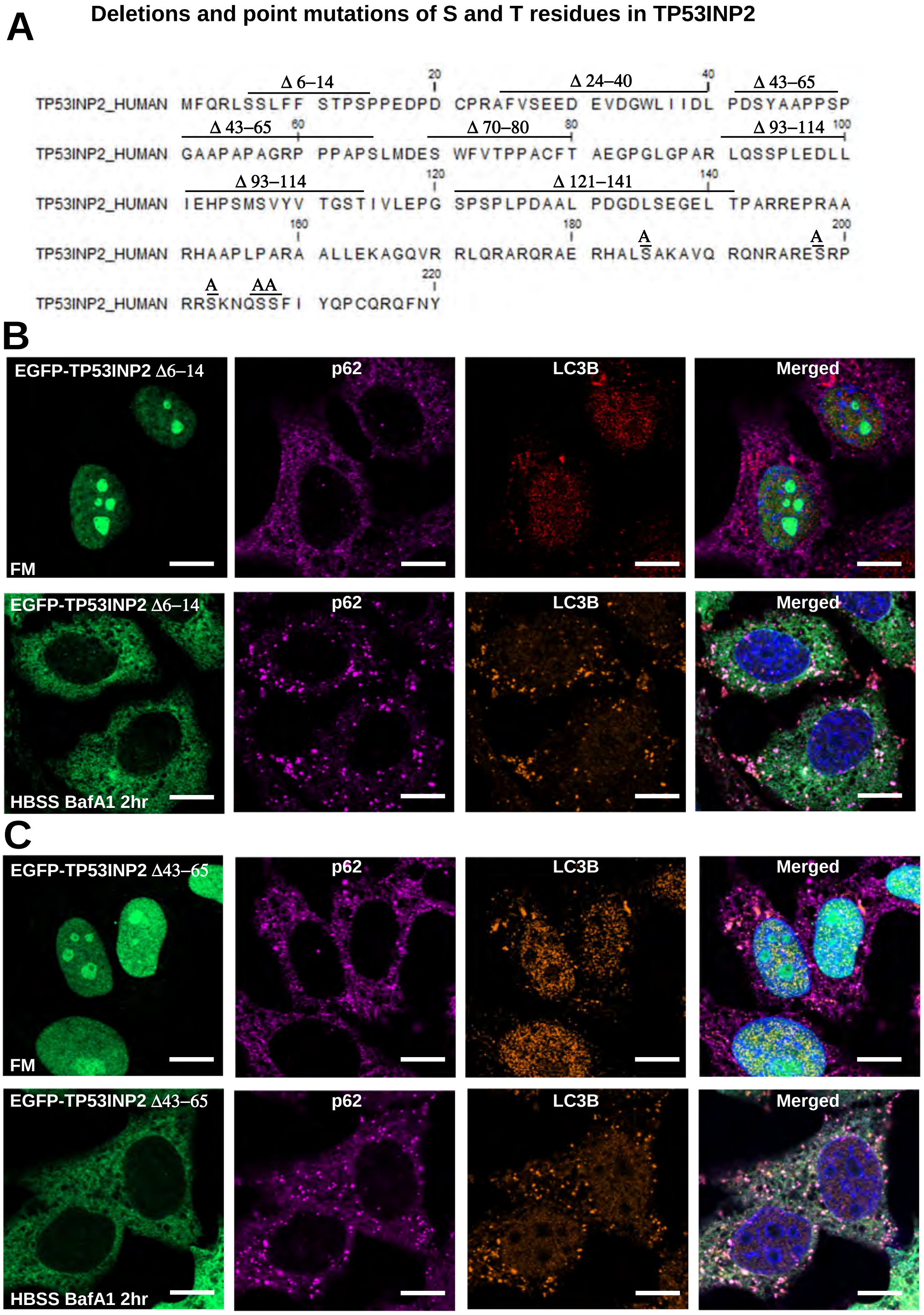

Journal of Cell Science • Supplementary information

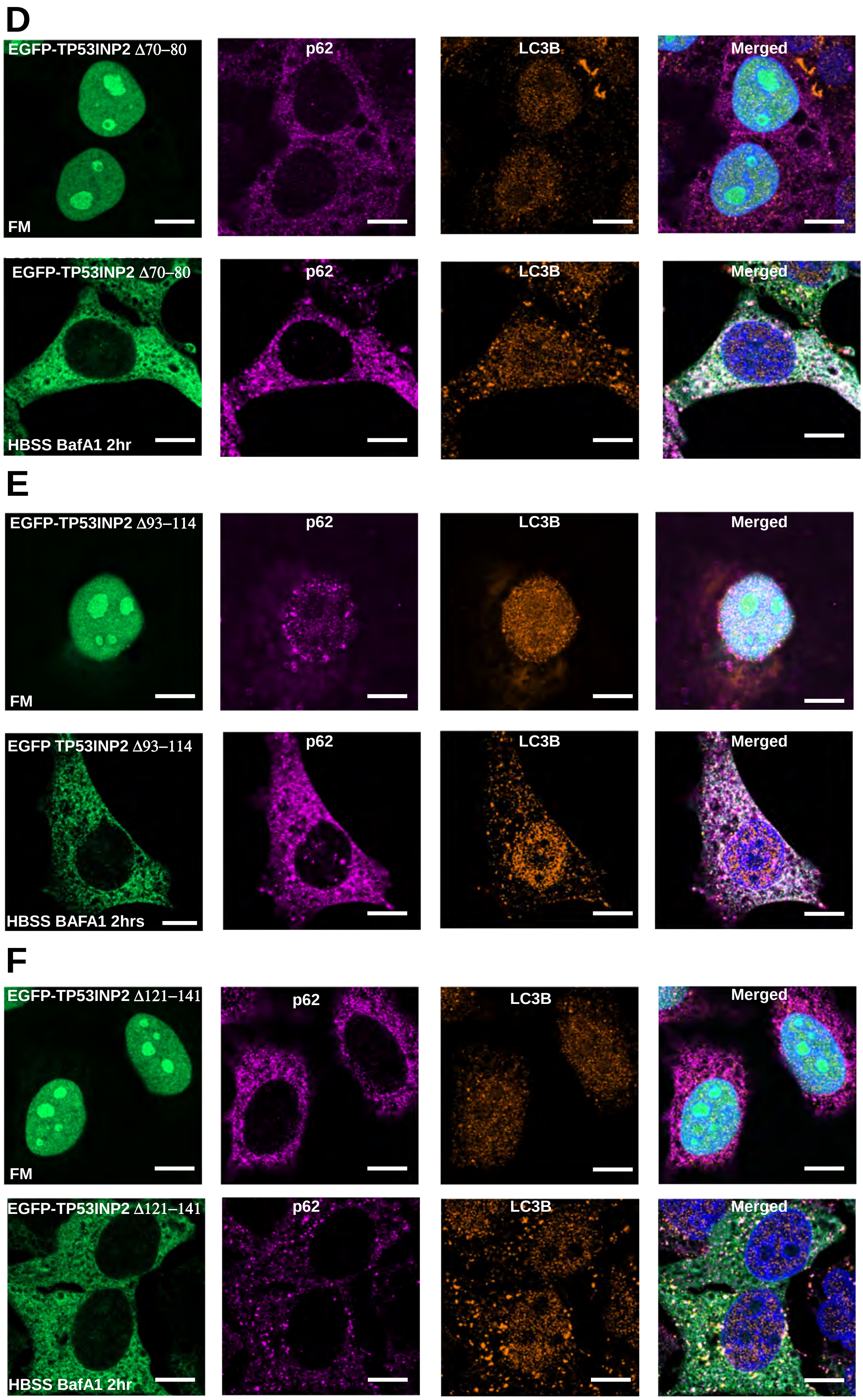
D
EGFP-TP53INP2 Δ70–80
p62
LC3B
Merged
FM
EGFP-TP53INP2 Δ70–80
p62
LC3B
Merged
HBSS BafA1 2hr
E
EGFP-TP53INP2 Δ93–114
p62
LC3B
Merged
FM
EGFP TP53INP2 Δ93–114
p62
LC3B
Merged
HBSS BAFA1 2hrs
F
EGFP-TP53INP2 Δ121–141
p62
LC3B
Merged
FM
EGFP-TP53INP2 Δ121–141
p62
LC3B
Merged
HBSS BafA1 2hr

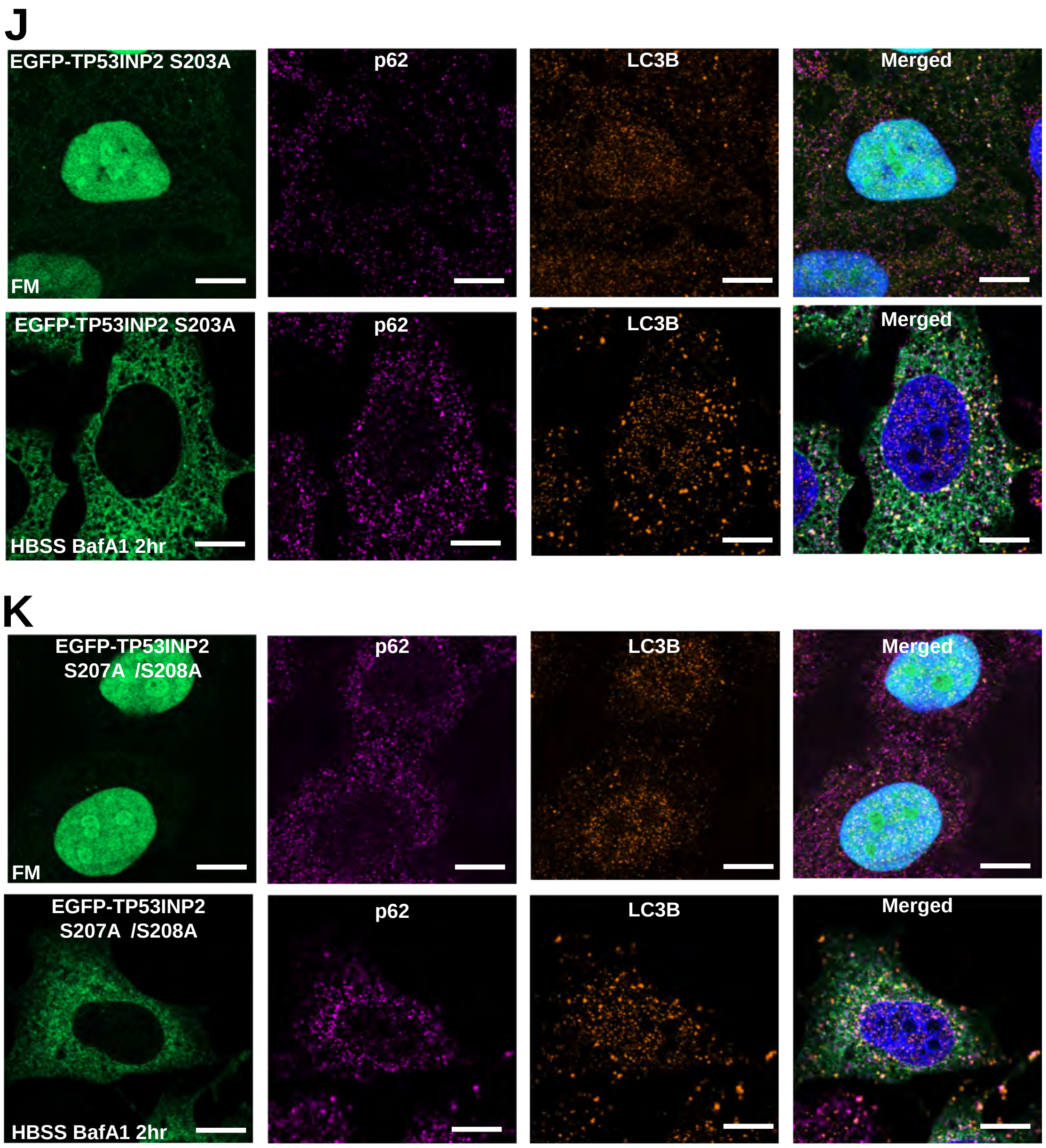

Fig. S5. Deletion of serine- and threonine residues does not affect the sub-cellular localization of TP53INP2.

(**A**) TP53INP2 amino acid sequence indicating mutated serine residues and the extension of systematic deletions done to identify essential serine or threonine residues. **(B-K)** Confocal fluorescence microscopy of HeLa FlpIn TP53INP2 KO cells stably expressing the indicated EGFP-TP53INP2 point mutant or deletion constructs, treated as indicated and stained with p62 and LC3B antibodies. Scale bars, 10 µm.

Journal of Cell Science • Supplementary information

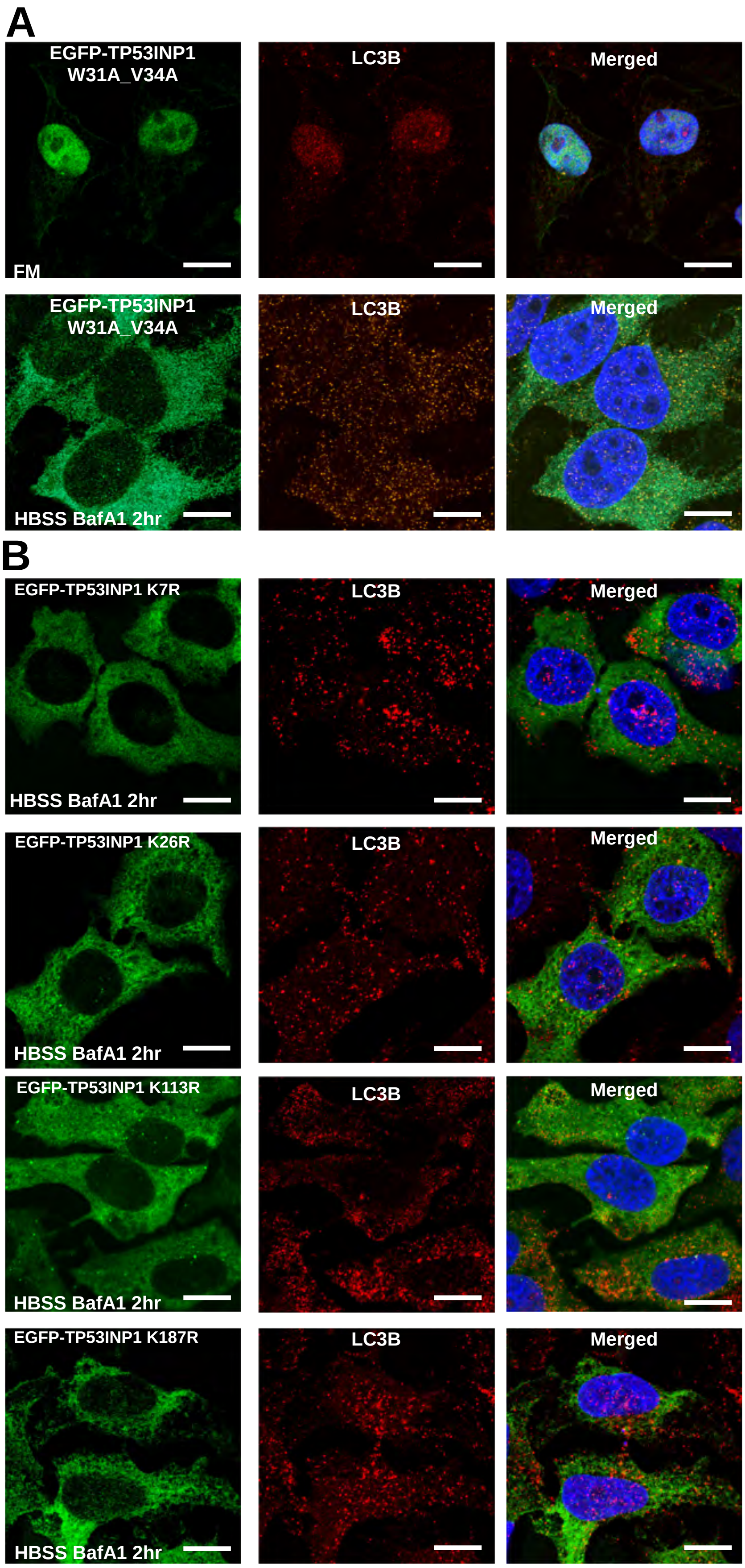
A
EGFP-TP53INP1 W31A_V34A
LC3B
Merged
FM
EGFP-TP53INP1 W31A_V34A
LC3B
Merged
HBSS BafA1 2hr
B
EGFP-TP53INP1 K7R
LC3B
Merged
HBSS BafA1 2hr
EGFP-TP53INP1 K26R
LC3B
Merged
HBSS BafA1 2hr
EGFP-TP53INP1 K113R
LC3B
Merged
HBSS BafA1 2hr
EGFP-TP53INP1 K187R
LC3B
Merged
HBSS BafA1 2hr

Fig. S6. The cytoplasmic localization of TP53INP1 upon starvation is independent of its LIR motif and lysine residues.

(A and B). HeLa FlipIn TP53INP1 KO cells stably reconstituted with indicated EGFP TP53INP1 constructs were grown in full medium or starved in HBSS for 2 hours. Then cells were fixed and immunostained with LC3B antibody for analysis with confocal microscopy. Scale bars, 10 μm.

BLOT TRANSPARENCY 1

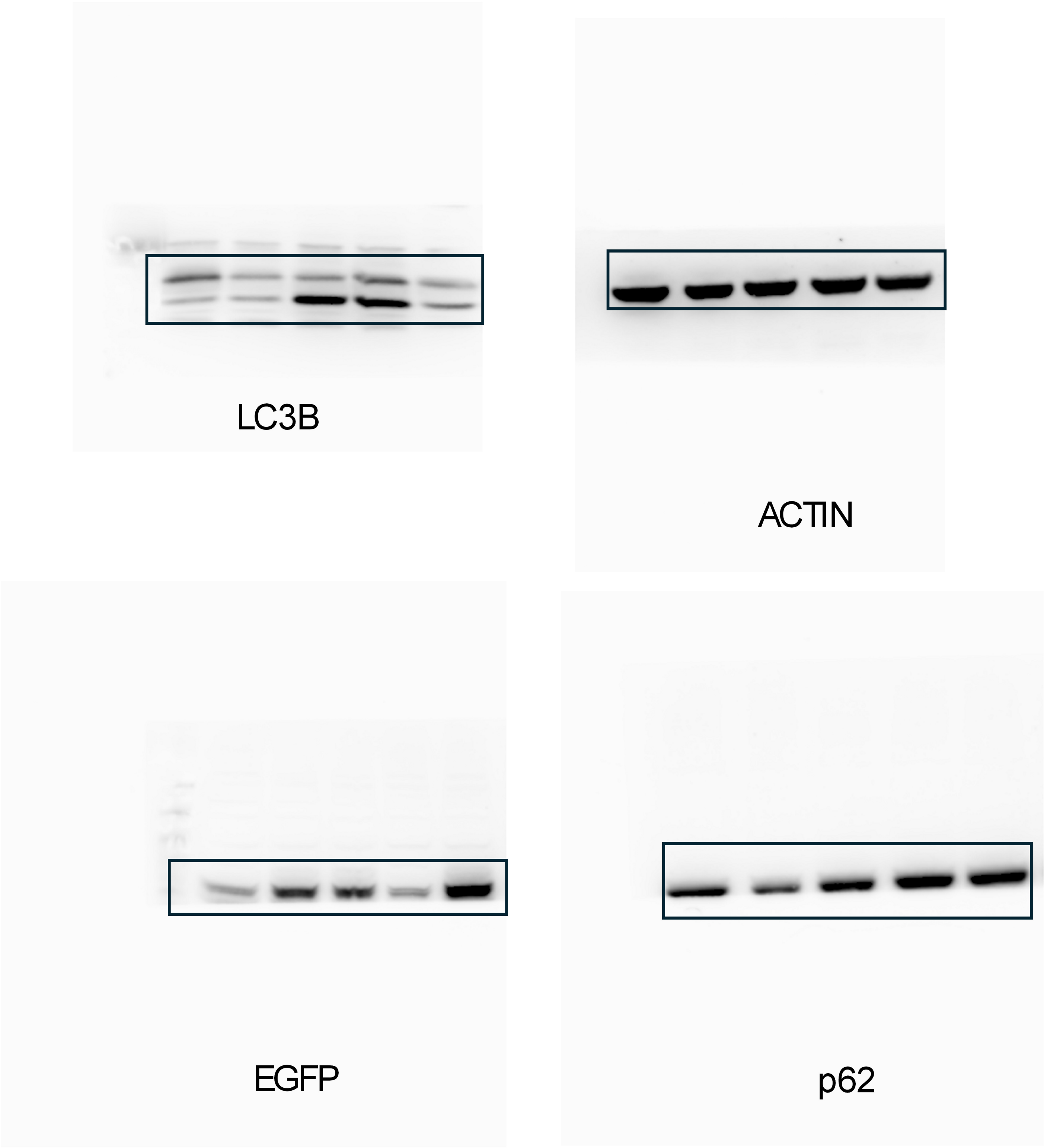

Figure 1B

Journal of Cell Science • Supplementary information

BLOT TRANSPARENCY 2

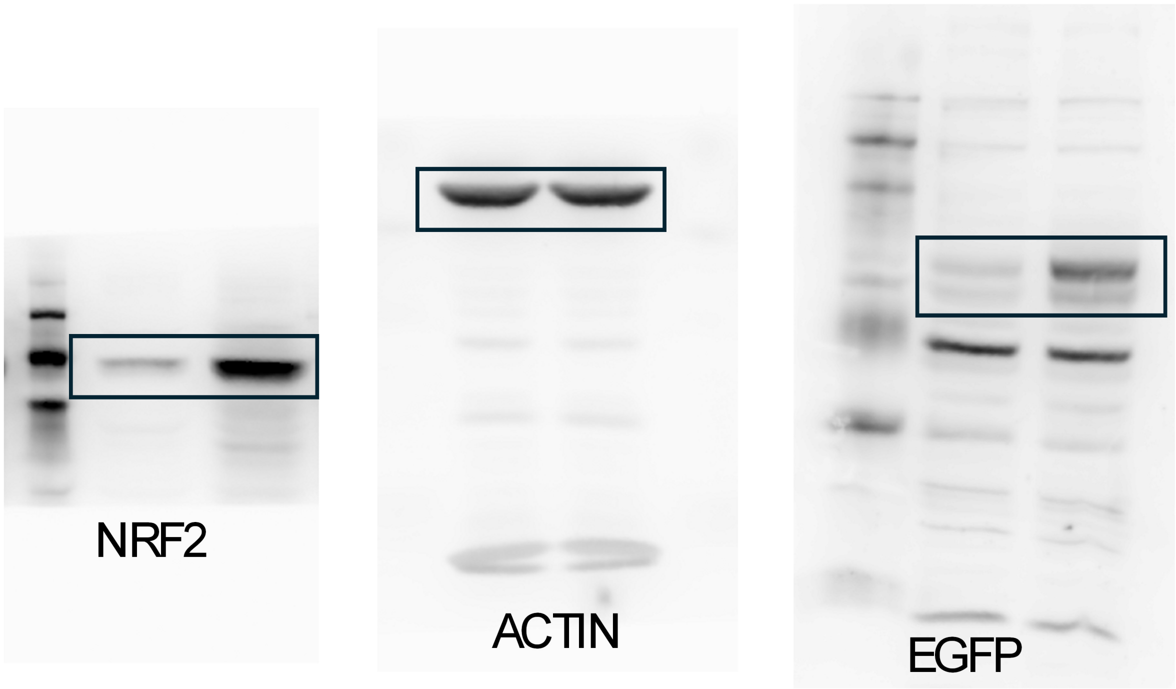

Figure 1E

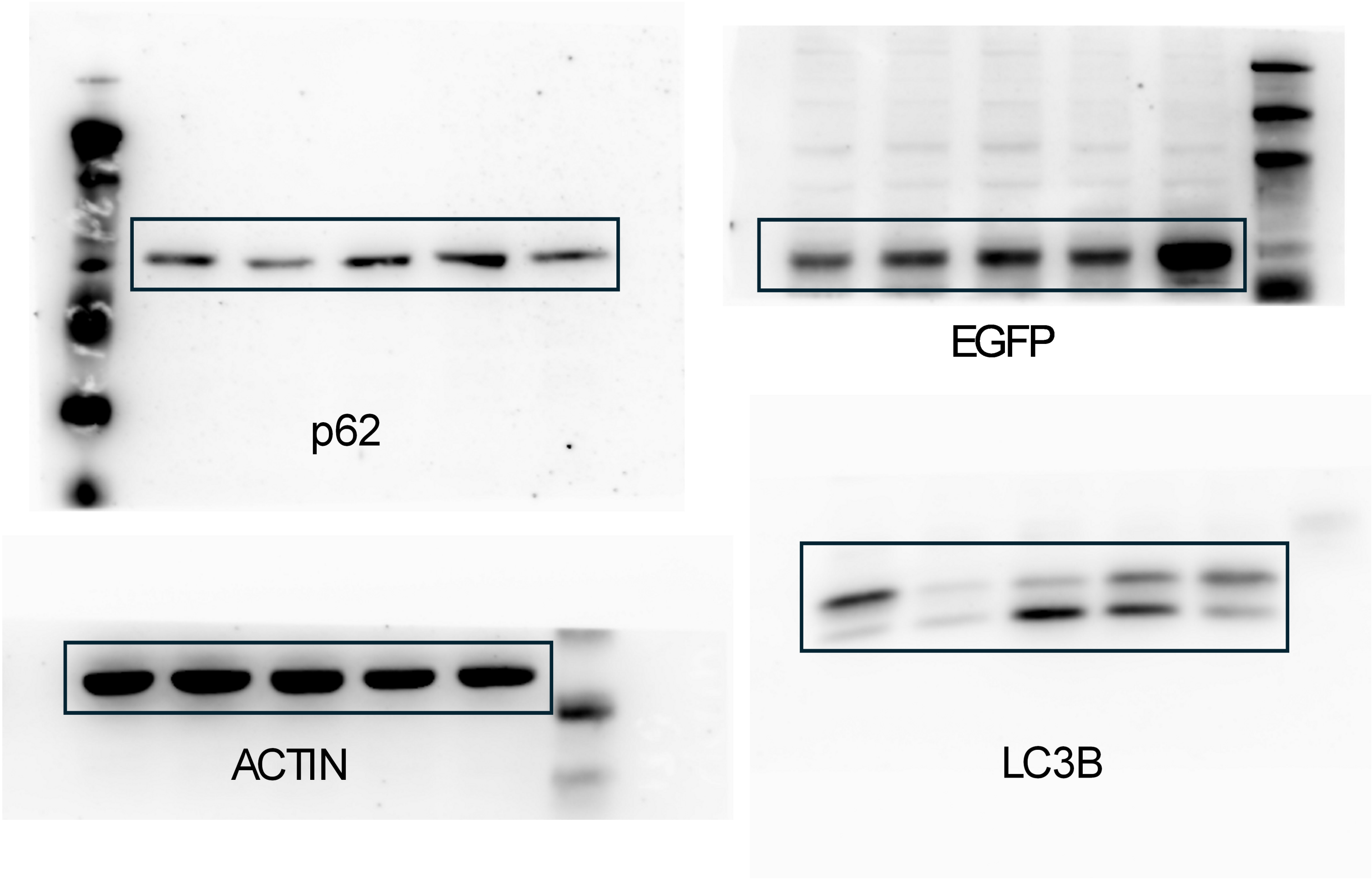

Figure 3C

BLOT TRANSPARENCY 3

Figure 5C

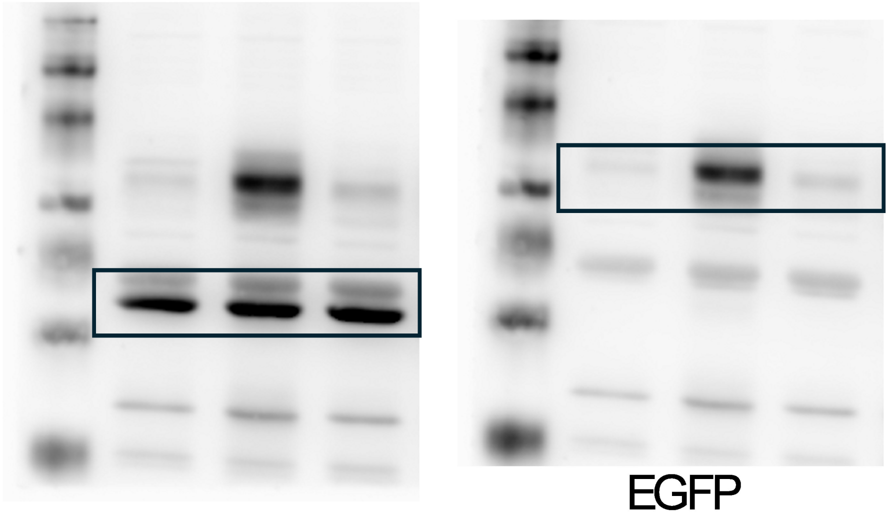

EGFP

ACTIN

Figure 5D

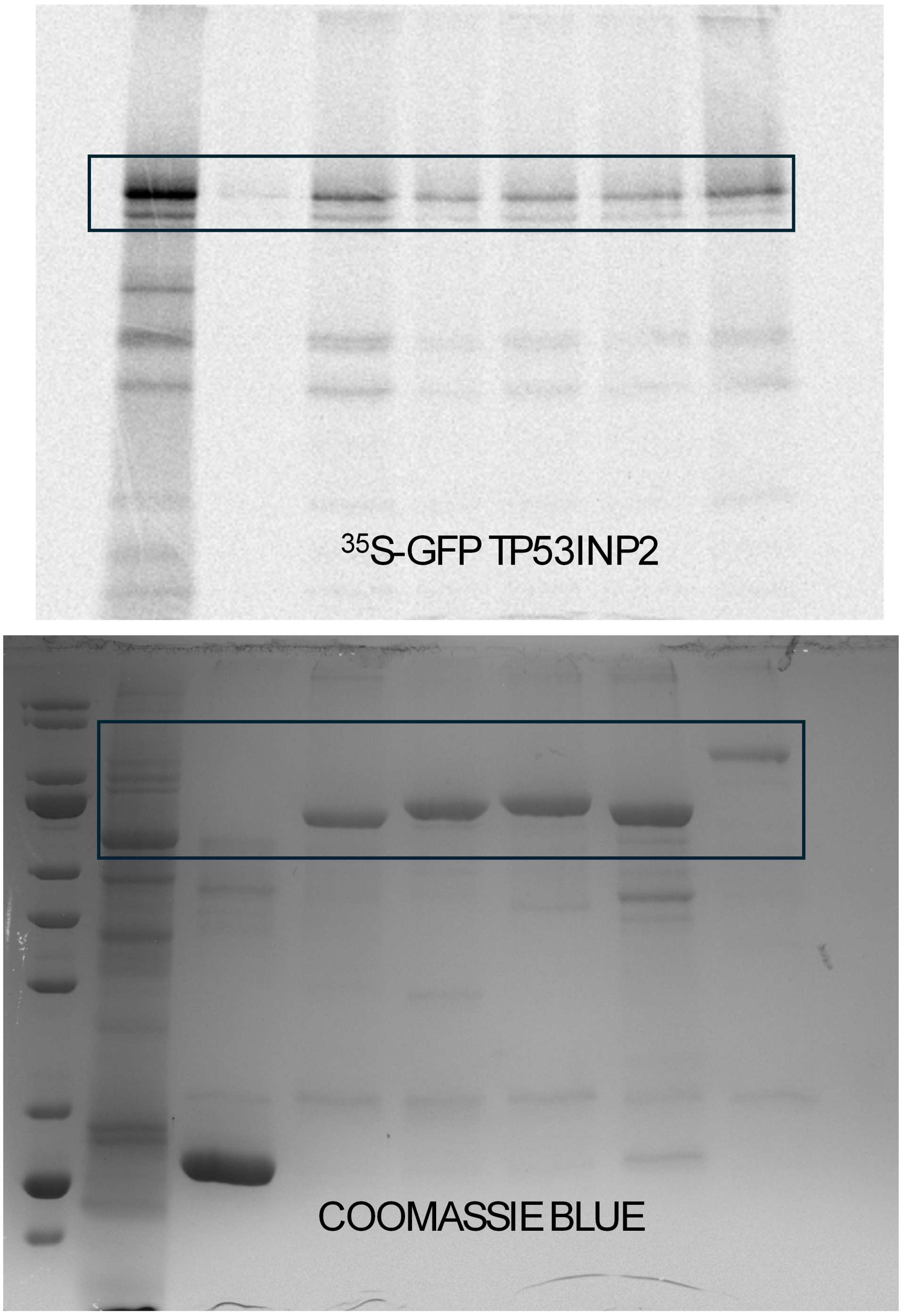

Journal of Cell Science • Supplementary information

BLOT TRANSPARENCY 4

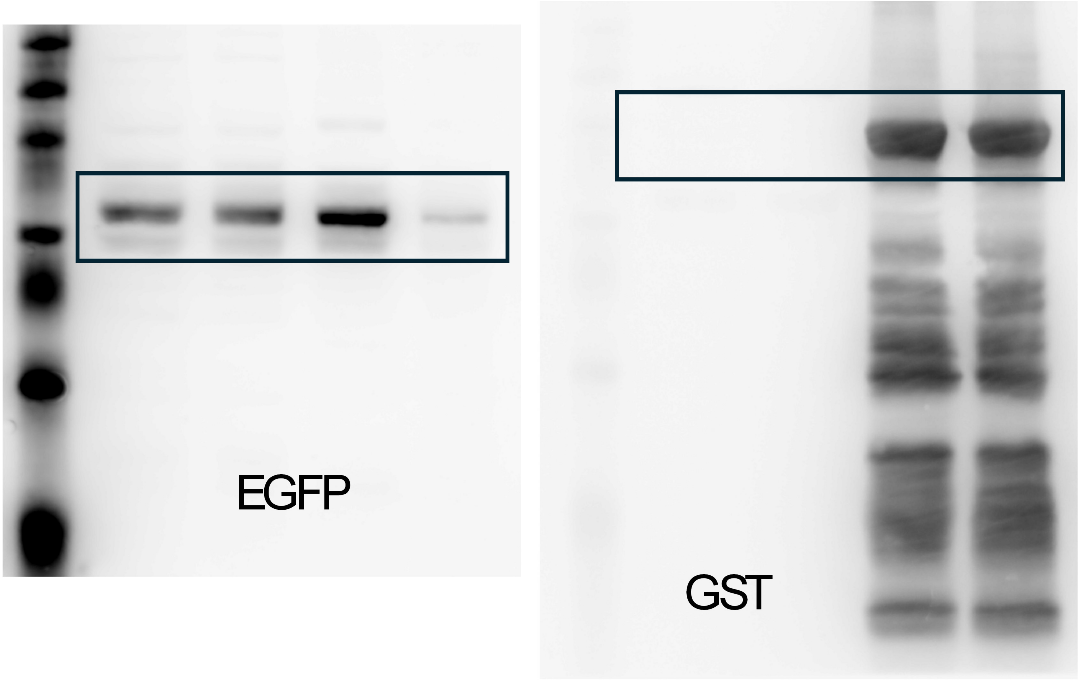

Figure 5E

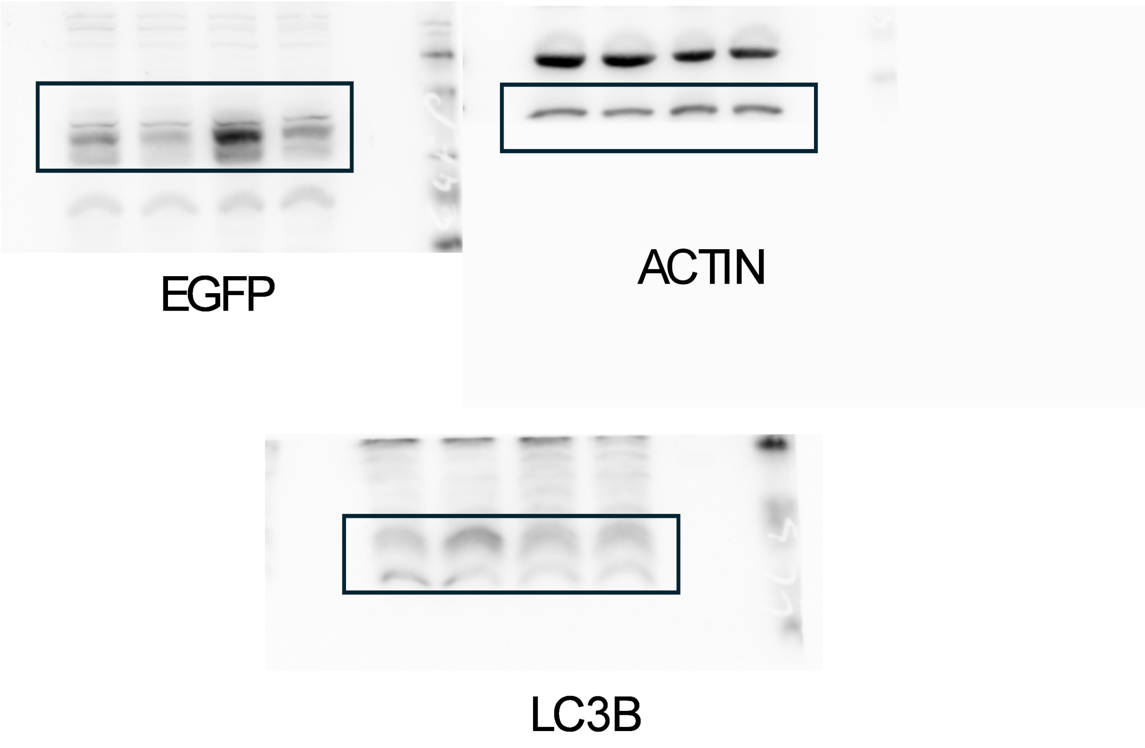

Figure 7C

Journal of Cell Science • Supplementary information

BLOT TRANSPARENCY 5

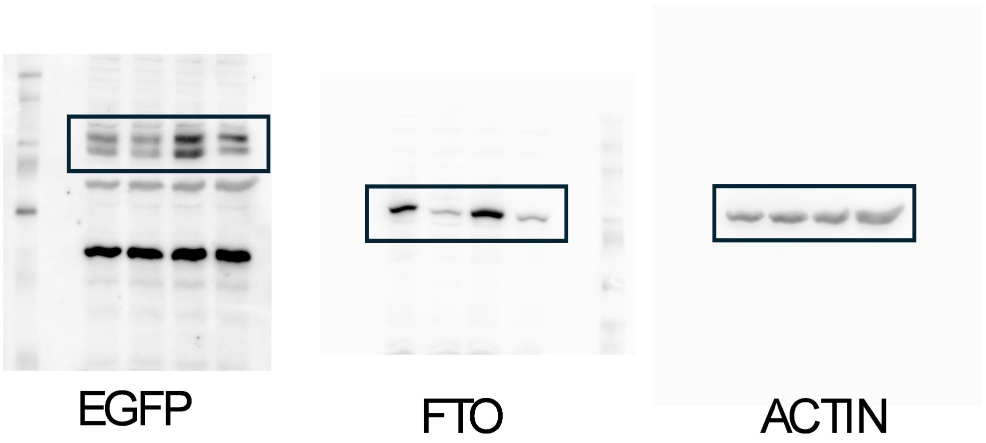

Figure 7E

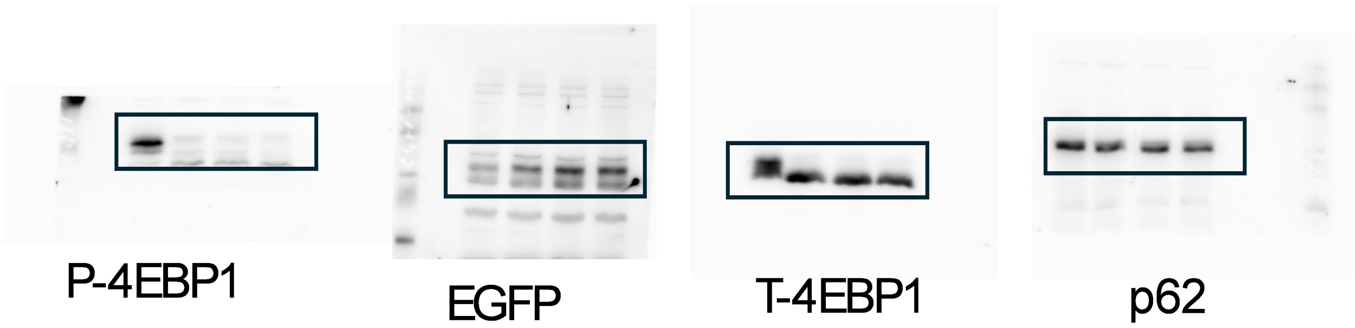

Figure 7G

Journal of Cell Science • Supplementary information

BLOT TRANSPARENCY 6

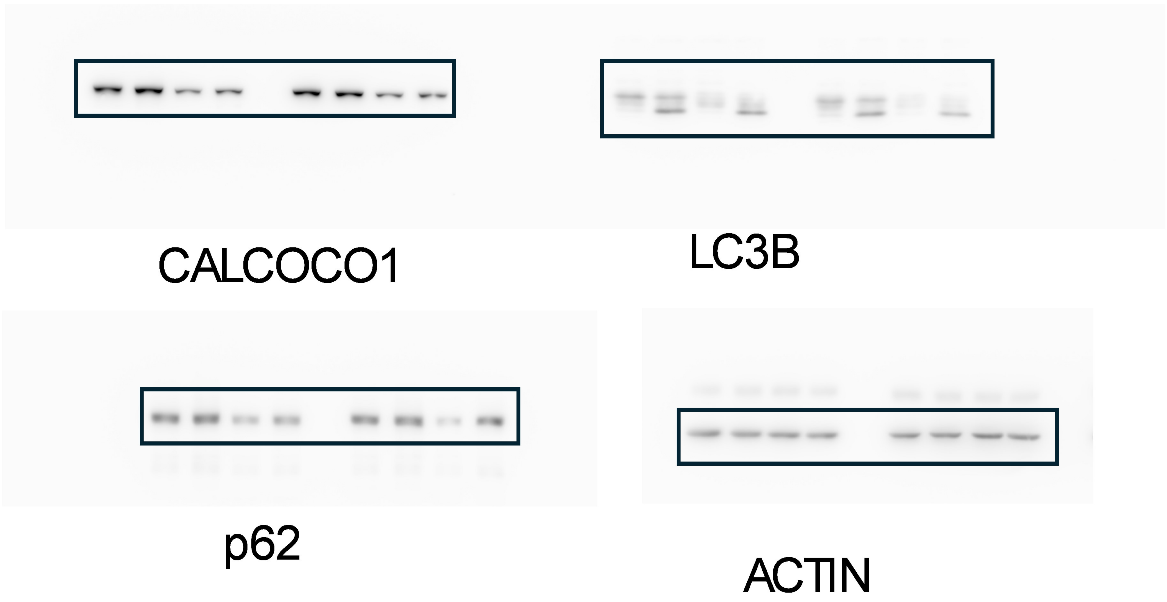

Figure S1B

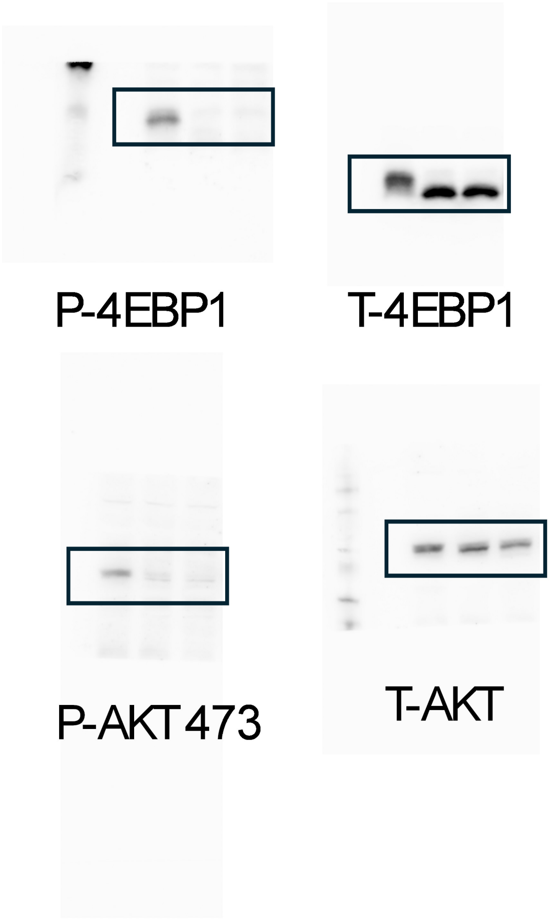

Figure S2B

Journal of Cell Science • Supplementary information

BLOT TRANSPARENCY 7

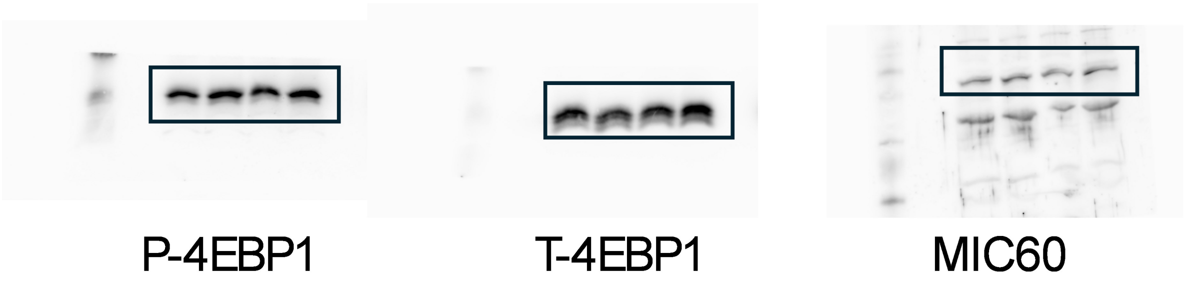

Figure S2D

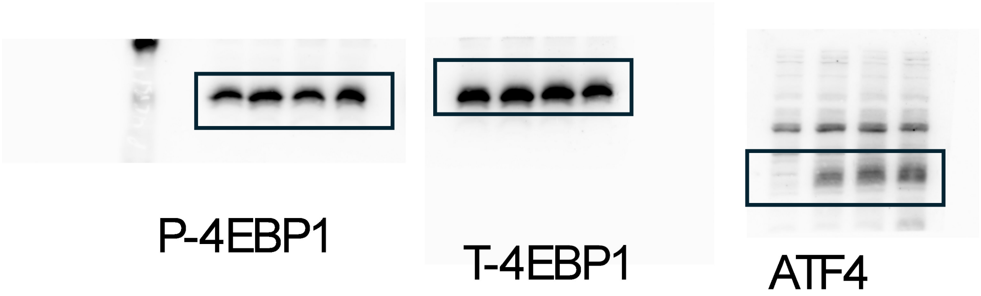

Figure S2F

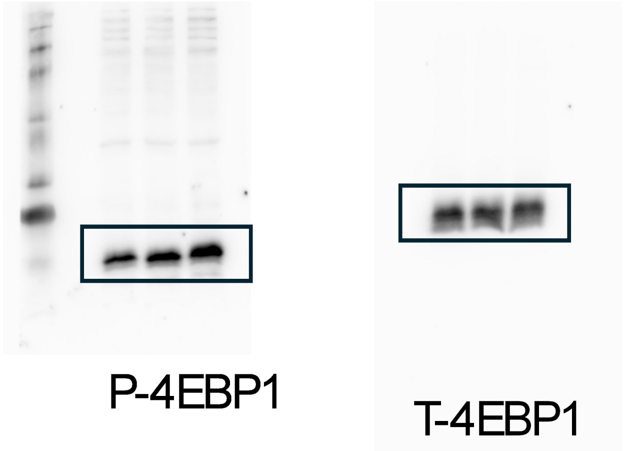

Figure S2J

Fig. S7. Blot transparency.

Journal of Cell Science • Supplementary information

Table S1. List of plasmids used in this study.

Gateway entry vectors

Plasmid	Source
pENTR -TP53INP2	(Sancho et al., 2012)
pENTR -TP53INP2 W35A/I38A	(Sancho et al., 2012)
pENTR -TP53INP2 K165R	This study
pENTR -TP53INP2 K187R	This study
pENTR -TP53INP2 K204R	This study
pENTR -TP53INP2 K165R/K187R/K204R	This study
pENTR -TP53INP2 E97K/D98K	(Sancho et al., 2012)
pENTR -TP53INP2 Δ24-40	This study
pENTR -TP53INP2 Δ189-211	This study
pENTR -TP53INP2 Δ201-204	This study
pENTR -TP53INP2 W35A/I38A/Δ189-211	This study
pENTR -TP53INP2 1-143	This study
pENTR -TP53INP2 1-165	This study
pENTR -TP53INP2 1-189	This study
pENTR -TP53INP2 1-211	This study
pENTR -TP53INP2 143-211	This study
pENTR -TP53INP2 143-165	This study
pENTR -TP53INP2 165-189	This study
pENTR -TP53INP2 189-211	This study
pENTR -TP53INP2 Δ6-14	This study

Journal of Cell Science • Supplementary information

pENTR -TP53INP2 Δ43-65	This study
pENTR -TP53INP2 46-220	This study
pENTR -TP53INP2 Δ70-80	This study
pENTR -TP53INP2 Δ93-114	This study
pENTR -TP53INP2 Δ121-141	This study
pENTR -TP53INP2 S185A	This study
pENTR -TP53INP2 S198A	This study
pENTR -TP53INP2 S207A/S208A	This study
pENTR -TP53INP2 S203A	This study
pENTR -TP53INP2 R216A/Q217A	This study
pENTR -TP53INP2 Q212A/P2173A	This study
pENTR -TP53INP1 1-231	This study

Gateway expression plasmids

Plasmid	Source
pDest EGFP-Flp-In-TP53INP2	This study
pDest EGFP-Flp-In-TP53INP2 W35A/I38A	This study
pDest EGFP-Flp-In-TP53INP2 K165R	This study
pDest EGFP-Flp-In-TP53INP2 K187R	This study
pDest EGFP-Flp-In-TP53INP2 K204R	This study
pDest EGFP-Flp-In-TP53INP2 K165R/K187R/K204R	This study
pDest EGFP-Flp-In-TP53INP2 E97K/D98K	This study

Journal of Cell Science • Supplementary information

pDest EGFP-Flp-In-TP53INP2 Δ24-40	This study
pDest EGFP-Flp-In-TP53INP2 Δ189-211	This study
pDest EGFP-Flp-In-TP53INP2 Δ201-204	This study
pDest EGFP-Flp-In-TP53INP2 Δ201-204	This study
pDest EGFP-Flp-In-TP53INP2 W35A/I38A/Δ189-211	This study
pDest EGFP-Flp-In-TP53INP2 46-220	This study
pDest EGFP-Flp-In-TP53INP2 1-211	This study
pDest EGFP -TP53INP2 1-143	This study
pDest EGFP -TP53INP2 1-165	This study
pDest EGFP -TP53INP2 1-189	This study
pDest EGFP -TP53INP2 1-211	This study
pDest EGFP -TP53INP2 143-211	This study
pDest EGFP -TP53INP2 143-165	This study
pDest EGFP -TP53INP2 165-189	This study
pDest EGFP -TP53INP2 189-211	This study
pDest EGFP-Flp-In-TP53INP2 Δ6-14	This study
pDest EGFP-Flp-In-TP53INP2 Δ43-65	This study
pDest EGFP-Flp-In-TP53INP2 Δ70-80	This study
pDest EGFP-Flp-In-TP53INP2 Δ93-114	This study
pDest EGFP-Flp-In-TP53INP2 Δ121-141	This study
pDest EGFP-Flp-In-TP53INP2 S185A	This study
pDest EGFP-Flp-In-TP53INP2 S198A	This study
pDest EGFP-Flp-In-TP53INP2 S207A/S208A	This study

pDest EGFP-Flp-In-TP53INP2 S203A	This study
pDest EGFP-Flp-In-TP53INP2 R216A/Q217A	This study
pDest EGFP-Flp-In-TP53INP2 Q212A/P2173A	This study
pDest EGFP-Flp-In-TP53INP1 1-231	This study

Additional plasmids

Plasmid	Source
Rev 1.4 EGFP	(Pankiv et al., 2010)
Rev 1.4 NES EGFP	(Pankiv et al., 2010)
Rev 1.4 TP53INP2 NES1 EGFP	This study
Rev 1.4 TP53INP2 NES1 EGFP	This study
PX459	Addgene #62988
pO44	Invitrogen

Journal of Cell Science • Supplementary information

Table S2. List of antibodies used for Western blot in this study.

Antibodies	Source	Identifier	Dilution
rabbit anti-LC3B	Novus	NB100-2220	1:1000
mouse anti-p62	BD Bioscience	610833	1:100
rabbit anti-CALCOCO1	Santa Cruz Biotech	sc–515670	1:1000
rabbit anti-GFP	Abcam	AB290	1:1000
rabbit anti-NRF2	Abcam	ab62352	1:1000
mouse anti-TOM20	Santa Cruz Biotech	ac-17764	1:1000
rabbit anti-4-EBP1	Cell Signaling	9644	1:1000
rabbit anti-4E-BP1 phospho S65	Cell Signaling	9451	1:1000
rabbit anti-AKT	Cell Signaling	9272	1:1000
rabbit anti-AKT phospho S473	Cell Signaling	9271S	1:1000
rabbit anti-MIC60	Novus	NB100-1919	1:1000
rabbit anti-ATF4	Santa Cruz Biotech	sc-200	1:1000
mouse anti-G3BP1	BD Bioscience	611126	1:200
rabbit anti-ACTIN	Sigma-Aldrich	A2066	1:1000
horseradish peroxidase-conjugated goat anti-mouse	BD Bioscience	554002	1:1000
goat anti-rabbit	BD Bioscience	554021	1:1000

Table S3. List of antibodies used for confocal fluorescence microscopy.

Antibodies	Source	Identifier	Dilution
rabbit anti-LC3B	Sigma-Aldrich	L7543	1:500
guinea pig anti-p62	Progen	GP62-C	1:2000
rabbit anti-fibrillarin	Santa Cruz Biotech	sc-25397	1:200
Alexa Fluor®555-conjugated goat anti-rabbit IgG	Life Technologies	A-21428	1:1000
Alexa Fluor®647-conjugated goat anti-guinea pig IgG	Life Technologies	A-21450	1:1000

Journal of Cell Science • Supplementary information
